# Bacterial Competition Systems Share a Domain Required for Inner Membrane Transport of the Bacteriocin Pyocin G from Pseudomonas aeruginosa

**DOI:** 10.1128/mbio.03396-21

**Published:** 2022-03-28

**Authors:** Iva Atanaskovic, Connor Sharp, Cara Press, Renata Kaminska, Colin Kleanthous

**Affiliations:** a Department of Biochemistry, University of Oxford, Oxford, United Kingdom; b Department of Zoology, University of Oxford, Oxford, United Kingdom; University of Nebraska-Lincoln

**Keywords:** pyocin, bacterial competition, antibiotic, *P. aeruginosa*, cell envelope, protein import

## Abstract

Bacteria exploit a variety of attack strategies to gain dominance within ecological niches. Prominent among these are contact-dependent inhibition (CDI), type VI secretion (T6SS), and bacteriocins. The cytotoxic endpoint of these systems is often the delivery of a nuclease to the cytosol. How such nucleases translocate across the cytoplasmic membrane of Gram-negative bacteria is unknown. Here, we identify a small, conserved, 15-kDa domain, which we refer to as the inner membrane translocation (IMT) domain, that is common to T6SS and bacteriocins and linked to nuclease effector domains. Through fluorescence microscopy assays using intact and spheroplasted cells, we demonstrate that the IMT domain of the Pseudomonas aeruginosa-specific bacteriocin pyocin G (PyoG) is required for import of the toxin nuclease domain to the cytoplasm. We also show that translocation of PyoG into the cytosol is dependent on inner membrane proteins FtsH, a AAA+ATPase/protease, and TonB1, the latter more typically associated with transport of bacteriocins across the outer membrane. Our study reveals that the IMT domain directs the cytotoxic nuclease of PyoG to cross the cytoplasmic membrane and, more broadly, has been adapted for the transport of other toxic nucleases delivered into Gram-negative bacteria by both contact-dependent and contact-independent means.

## INTRODUCTION

Bacteria deploy various contact-dependent and -independent competition systems to compete for space and resources (1). These systems deliver toxic effectors to the cell envelope or the cytoplasm of bacterial competitors. While the mechanism of effector delivery varies between different competition systems, effector structures and killing mechanisms can be conserved (2). Often, competition system effectors are nucleases that get transported across the cell envelope to degrade cytoplasmic nucleic acids. These folded proteins cross the multilayered cell envelope via poorly understood translocation mechanisms.

Bacteriocins of Gram-negative bacteria are protein antibiotics deployed as weapons for contact-independent bacterial competition. Bacteriocins have the potential to be developed into antibiotics for the treatment of infections resistant to conventional antimicrobials (3). Bacteriocins can kill bacterial cells via different mechanisms: by forming pores in the inner membrane; by inhibiting peptidoglycan biosynthesis through lipid II degradation; and by DNA or RNA degradation. Bacteriocin-producing strains are immune to their own bacteriocins due to the production of immunity proteins that inhibit the bacteriocin’s killing activity. Sensitive strains do not produce the immunity protein or produce an immunity protein specific for a different bacteriocin but nevertheless have the appropriate translocation machinery through the cell envelope. In the case of nuclease bacteriocins, which penetrate the cytoplasm to degrade nucleic acids, this translocation machinery spans both the outer and the inner membrane (4).

Pyocins are bacteriocins that target and kill Pseudomonas aeruginosa. Pyocin G (PyoG) is a nuclease pyocin active against P. aeruginosa clinical isolates (5). It is composed of an unstructured N terminus followed by a receptor binding domain (5), a conserved middle domain that is essential for its killing activity (6), and a cytotoxic nuclease domain that binds to the PyoG immunity protein (ImG) (7). The receptor binding domain is required for outer membrane translocation of PyoG (5). The role of the conserved central domain is unknown, but the fact that it is absent from bacteriocins with periplasmic targets and present in bacteriocins that cleave nucleic acids (Pfam domain PF06958.7) suggests that it has a role in inner membrane transport (8). Here, we show that this domain is required for PyoG translocation to the cytosol.

PyoG enters the periplasm by binding and translocating through Hur in the outer membrane (5). Hur is a 22-stranded β-barrel TonB-dependent transporter (TBDT) that ordinarily transports hemin into the cell following engagement with TonB1 in the inner membrane, in conjunction with the stator complex ExbB-ExbD and the proton motive force (PMF). TonB1 activates hemin transport following association with Hur’s periplasmically located TonB box epitope. PyoG parasitizes hemin uptake and in so doing delivers its own TonB box to the periplasm, which engages with TonB1, allowing the toxin to be pulled into periplasm.

TonB-dependent outer membrane translocation of pyocins is well understood (5, 9, 10). In contrast, how these toxins translocate from the periplasm to the cytoplasm is unknown. In the case of nuclease colicins, which are E. coli-specific bacteriocins, inner membrane transport requires both the ATPase and protease functions of FtsH (11). FtsH is a hexameric AAA+ ATPase/protease in the inner membrane, where it is involved in protein quality control (12). FtsH proteolytically processes nuclease colicins as they enter the cytoplasm (13). More recently, FtsH has also been linked to the killing activity of PyoG (5), demonstrating a broader involvement in bacteriocin uptake.

In the present work, we address the poorly understood area of bacteriocin transport across the inner membrane. Using a newly developed import assay, whereby the uptake of fluorescently labeled PyoG into P. aeruginosa spheroplasts is monitored, we map the requirements for pyocin transport to the cytoplasm. Using this assay, we demonstrate that a small domain found in all nuclease bacteriocins as well as other bacterial competition systems is absolutely required for transport.

## RESULTS AND DISCUSSION

### Inner membrane translocation of nucleases in bacterial competition systems is associated with a ubiquitous domain.

**FIG 1:**
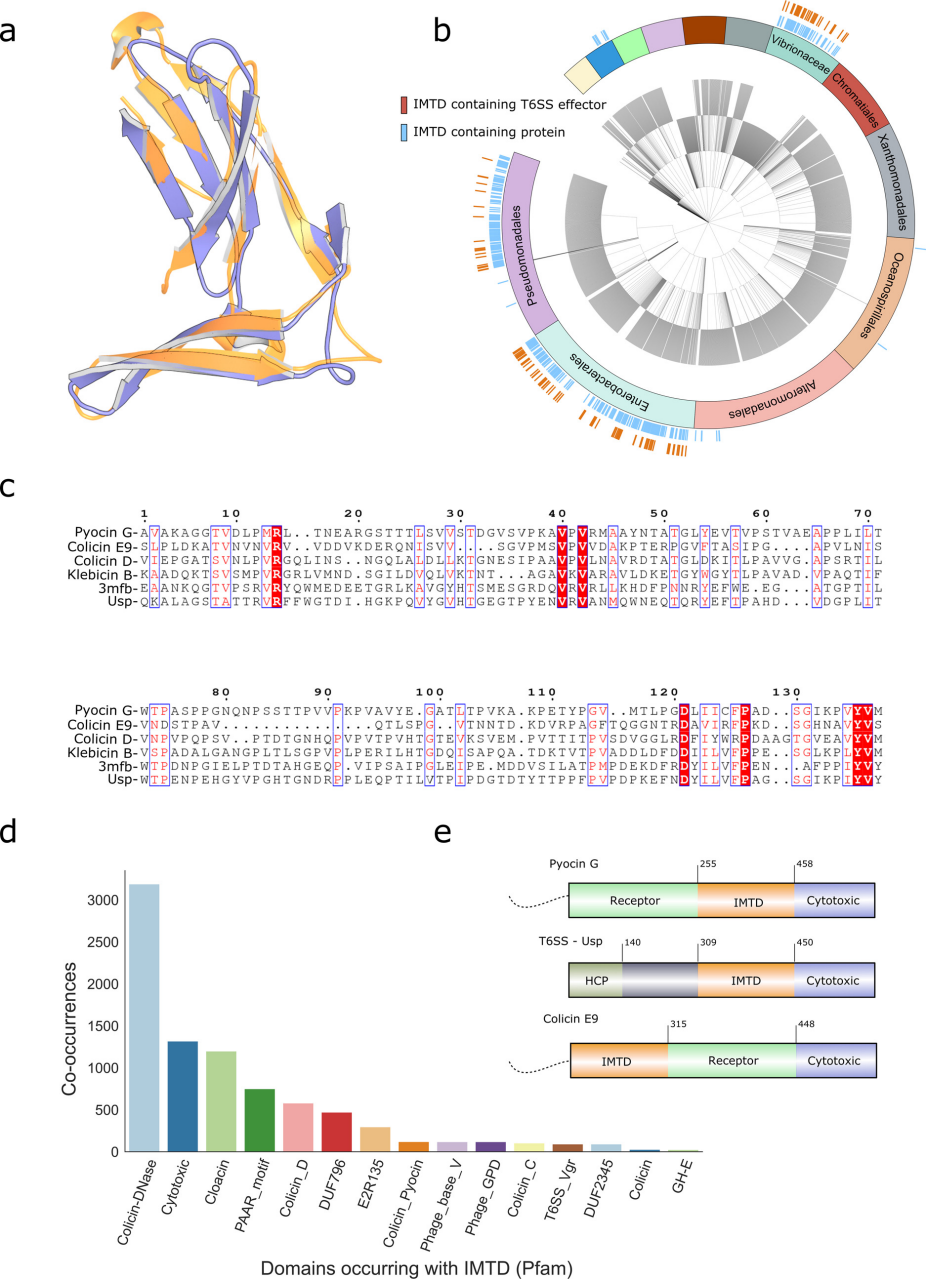
IMT domain is a conserved structural domain found in multiple orders within *Gammaproteobacteria* and associated with multiple toxin systems. (A) Structure of the conserved beta-sheets of the IMT domain from colicin D (blue, PDB entry 5ZNM) and a T6SS effector (gold, PDB entry 3MFB). (B) Taxonomy of *Gammaproteobacteria* within uniref100. Ticks indicate the presence of at least 1 IMT domain-containing protein (blue) and predicted T6SS effector (gold). IMT domain proteins are prevalent in *Gammaproteobacteria* though restricted to certain orders (unlabeled taxa are *Cellvibrionales*, gray; *Thiotrichales*, peach; *Pasteurellaceae*, purple; *Legionellales*, green; *Aeromonadales*, blue; *Methylococcales*, yellow). (C) Alignment of the IMT domain from bacterial toxins including colicins and other nuclease bacteriocins (colicin E9, colicin D, klebicin B, pyocin S2) and T6SS effectors (ECA1669 protein from Erwinia carotovora, PDB entry 3MFB; Usp, uropathic specific protein). (D) Pfam domains that cooccur in proteins with IMT domain. IMT domain is found in proteins that contain numerous toxin effectors or structural domains. Colicin-DNase, common HNH nuclease found in DNase bacteriocins and T6SS effectors; cytotoxic, rRNase toxin found in bacteriocins and T6SS effectors; Colicin_D, tRNase found in bacteriocins and T6SS effectors; PAAR motif, cloacin, DUF796 (T6SS_HCP), structural motif found in T6SS effectors; E2R135, receptor domain for certain colicins; Colicin_Pyocin, a domain found in the immunity proteins for many DNase bacteriocins. Phage_base_V/Phage_GPD, phage/T6SS structural proteins; Colicin_C, tRNase found in bacteriocins and T6SS effectors; T6SS_Vgr, structural domain of the T6SS; DUF2345, domain of unknown function associated with the T6SS; colicin, pore-forming domain found in many colicins, all proteins identified with this domain had similarity to colicin B, GH-E, HNH family of nucleases. (E) The domain organization of PyoG (5). For comparison, the position of the IMT domain is shown for a T6SS nuclease effector (Usp) and a nuclease colicin (ColE9). The unstructured N terminus of bacteriocins is represented with a dotted line.

Using bioinformatics, we have previously identified a highly conserved bacteriocin domain that is absent from pore-forming bacteriocins but associated exclusively with competition systems that transport nucleases to the cytoplasm (8). We term this domain the inner membrane translocation (IMT) domain. The IMT domain is annotated in the PFAM database as the pyocin-S domain (PF06958.7). The main structural features of the domain are two antiparallel β-sheets that give the domain an L-shape (PDB entry 5ZNM; Fig. 1A). Using a more extensive informatics search, we found the IMT domain across several orders of *Gammaproteobacteria* (Fig. 1B). Surprisingly, apart from being conserved among nuclease bacteriocins, this domain is also found in type 6 secretion system (T6SS) effectors (Fig. 1C). Even though the sequences of T6SS effectors and nuclease bacteriocins are divergent (Fig. 1C), the structural overlap is remarkable (Fig. 1A) and key residues align in both sequence and structure (Fig. 1C). These toxins, like nuclease bacteriocins, translocate across the inner membrane to kill competitors. Unlike bacteriocins, however, which are diffusible toxins, T6SS effectors are contact dependent and delivered by a contractile needle that punctures the outer membrane. We found several characteristic PFAM domains of these toxin systems cooccur with the IMT domain (Fig. 1D), the HNH nuclease domain found in DNase bacteriocins and T6SS effectors, and the rRNase and tRNase bacteriocin domains. A previous analysis of nuclease bacteriocins identified the IMT domain as a conserved feature of nuclease bacteriocins (8). Of all UniProt proteins we identified that contained the IMTD, 15.2% were predicted to be possible T6SS effectors based on T6SS species domains and homology to proteins in the SecReT6 database (14). To identify how often the IMTD is present in T6SS effectors, we scanned the SecRet6 database for all T6SS effectors that use the Colicin-DNase toxin domain. We found that 97.6% of proteins also contained the IMTD, suggesting that the IMTD is necessary in these T6SS effectors. However, not all nuclease toxin systems appear to utilize the IMTD. Contact-dependent inhibition systems can deliver rRNase toxins similar to colicins but do not encode the IMTD, suggesting they have evolved an alternative mechanism to cross the inner membrane. Nuclease domains are strongly associated with the IMTD (presence of nuclease domain with IMTD: Colicin-DNase, 57.5%; ColD tRNase, 62.9%; ColE3 tRNase, 59.3%). We conclude that the IMT domain is found in competition system effectors that express their cytotoxic activity in the cytoplasm of target cells regardless of the means by which the toxin initially penetrates the outer defenses of the Gram-negative bacterium.

### Inner membrane translocation of PyoG requires the IMT domain.

**FIG 2:**
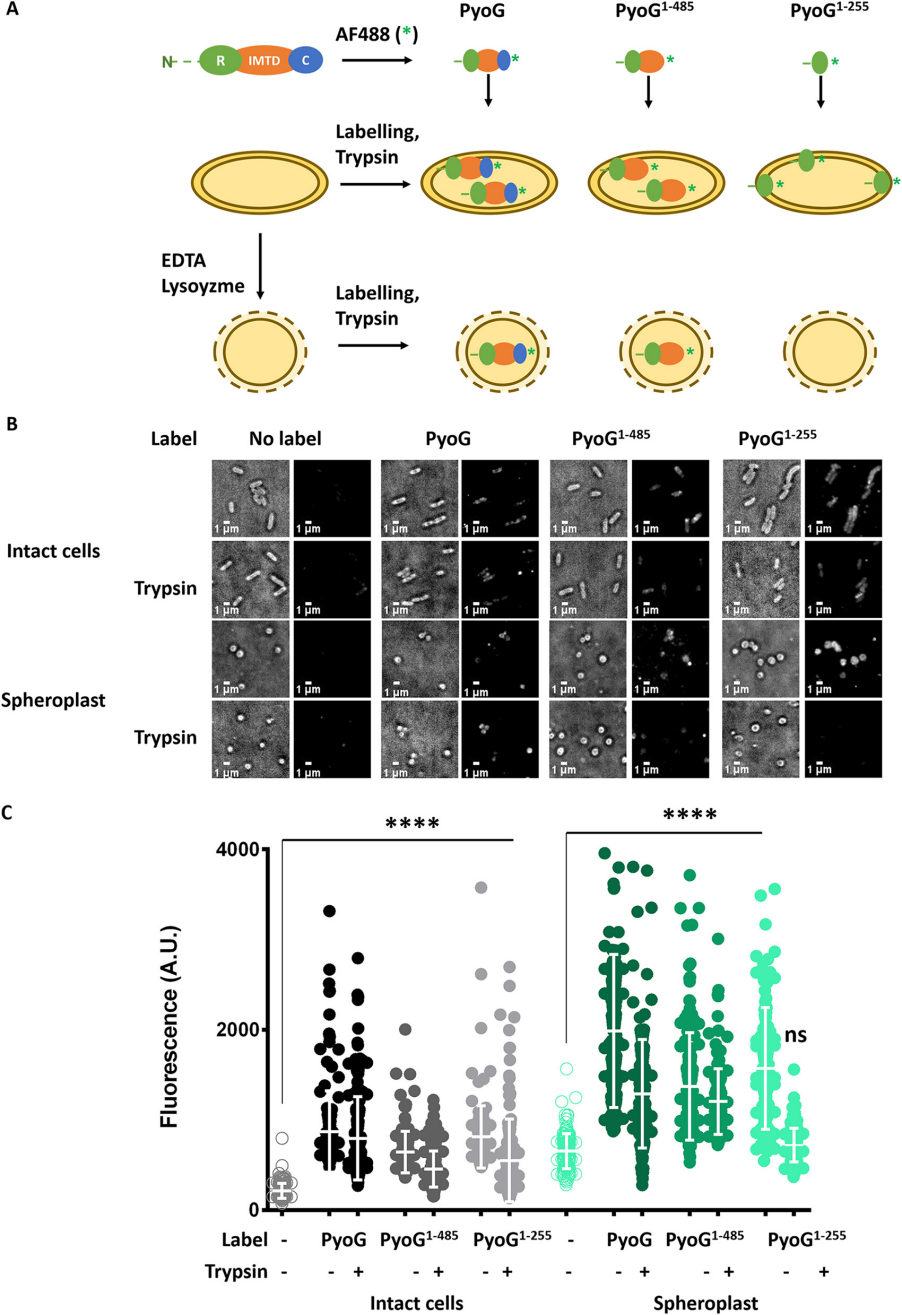
Import and localization of fluorescent PyoG constructs. (A) A fluorescence microscopy experiment setup, used for localization of PyoG constructs in P. aeruginosa cells. All constructs are conjugated to AF488 (represented with a green asterisk) via a C-terminal cysteine. Intact cells, or spheroplasts generated by lysozyme/EDTA treatment, are exposed to 2 μM fluorescent PyoG. Unbound and untranslocated pyocin constructs are removed by trypsin treatment. (B) Representative micrographs before and after trypsin treatment are shown. All snapshots were adjusted to the same contrast value. Tested constructs are full-length PyoG, PyoG^1–485^ lacking the cytotoxic domain, and PyoG^1–255^ lacking the cytotoxic domain and the IMT domain. (C) Average fluorescence intensities were measured for 150 cells per condition. Means from three biological replicates with standard deviations are shown. Fluorescence intensities for labeled and trypsin-treated groups under each condition are compared to the unlabeled control. ****, *P* value below 0.0001 in the Kruskal-Wallis Test; ns represents no significant difference or lack of fluorescent labeling.

Having established that the IMT domain is associated with the killing activity of toxic nucleases in bacteria, we sought to test its requirements for import of PyoG into P. aeruginosa. PyoG is a 640-amino-acid toxin comprised of an N-terminal receptor-binding domain that engages Hur and enables transport to the periplasm and a C-terminal nuclease domain that elicits cell death (5). Between them is the IMT domain, which contains residues 256 to 485 (Fig. 1E). We developed a fluorescence microscopy assay to dissect the involvement of the IMT domain in transport. Pyocins can be readily conjugated with fluorescent dyes and used for labeling live P. aeruginosa cells that express components of the pyocin translocation machinery in microscopy experiments. Trypsin protection is then used to distinguish imported from surface-bound molecules (9, 10). We generated different PyoG constructs, each containing a unique cysteine at the C terminus of the construct that was conjugated to Alexa Fluor (AF) 488, and assessed the ability of each construct to be imported in intact cells and spheroplasts (Fig. 2A). Spheroplasts were generated by lysozyme/EDTA treatment that permeabilizes the outer membrane and peptidoglycan layer (15–17). After labeling with fluorescent pyocin, cells were exposed to trypsin to remove any surface-exposed pyocin that was not translocated across the cell envelope. In intact cells, the outer membrane is not permeable to trypsin (9, 10). Therefore, in intact cells translocation across the outer membrane is sufficient to protect a pyocin construct from degradation with trypsin. In spheroplasts, the periplasm is exposed to trypsin (15), and the pyocin construct must translocate across the inner membrane to be protected from degradation by trypsin. Residual fluorescence after trypsin treatment is therefore an indication of outer membrane, and potentially inner membrane, transport in intact cells. In the case of spheroplasts, it is an indication of inner membrane transport. To test if nontranslocated pyocin gets degraded by trypsin under the conditions of our experiments, a pyocin S2-GFP chimera was used (see Fig. S1A in the supplemental material). This construct is composed of the pyocin S2 receptor binding domain (residues 1 to 209) translationally fused to green fluorescent protein (GFP). GFP acts as a plug blocking the import of the pyocin (10). We used this construct as a control in place of the equivalent PyoG construct fused to GFP (PyoG^1–255^), which could not be overexpressed in bacteria. Trypsin treatment removed the PyoS2-GFP fluorescent signal entirely in both intact cells and spheroplasts (Fig. S1A and B). A further control was undertaken to ascertain if the outer membrane translocation step can be bypassed using spheroplasts. Δ*hur* strain, a PAO1 transposon mutant that lacks the PyoG receptor (5), was exposed to fluorescent PyoG. In prior work, intact cells of this mutant were not labeled with the pyocin, since Hur is essential for binding of PyoG to the surface of P. aeruginosa cells (5). Generation of spheroplasts was sufficient to allow Δ*hur* strain labeling with fluorescent PyoG. Additionally, PyoG was protected from degradation by trypsin in Δ*hur* spheroplasts (Fig. S1D and E). Therefore, the Hur-dependent outer membrane translocation step can be bypassed under these tested experimental conditions, confirming that P. aeruginosa spheroplasts could be used to study inner membrane translocation.

10.1128/mbio.03396-21.1 FIG S1 S2-GFP is not trypsin protected in P. aeruginosa PAO1 intact cells and spheroplasts. (A) S2-GFP is comprised of the S2 N-terminal domain (S2^1-209^), translationally fused to GFP. GFP acts as a plug that blocks translocation of S2 across the cell envelope (10). This construct is used to test if surface-exposed pyocin gets degraded by trypsin in the fluorescence microscopy experiment described in Fig. 1. (B and C) Intact cells, or spheroplasts generated with lysozyme/EDTA treatment, are exposed to 2 μM S2-GFP. The S2-GFP fluorescent signal is removed by trypsin in both intact cells and spheroplasts. This indicates that untranslocated pyocin is removed by trypsin under tested experimental conditions. (D and E) Hur, the outer membrane receptor of PyoG, is not required for PyoG import into spheroplasts. Therefore, the outer membrane translocation step is bypassed in spheroplasts, which enables specific detection of inner membrane transport. Intact PAO1 Δ*hur* cells are not labeled with the pyocin, since Hur is required for binding of PyoG to the surface of P. aeruginosa cells (5). Generation of spheroplast with lysozyme/EDTA treatment is sufficient to allow Δ*hur* labeling with fluorescent PyoG. (B and D) Representative micrographs before and after trypsin treatment are shown. All snapshots were adjusted to the same intensity scale. (C and E) Average fluorescence intensities were measured for 150 cells per condition. Means from three biological replicates with standard deviations are shown. Fluorescence intensities for labelled and trypsin treated groups under each condition are compared to the unlabeled control. ****, *P* value below 0.0001 in the Kruskal-Wallis test, and ns represents no significant difference or lack of fluorescent labelling. Download FIG S1, TIF file, 1.9 MB. Copyright © 2022 Atanaskovic et al. 2022 Atanaskovic et al. https://creativecommons.org/licenses/by/4.0/ This content is distributed under the terms of the Creative Commons Attribution 4.0 International license.

The cellular localizations of the following PyoG constructs were tested: full-length PyoG, PyoG^1–485^, which lacks the cytotoxic domain, and PyoG^1–255^, which lacks the cytotoxic and IMT domains (Fig. 2A). Constructs were expressed and purified as previously described (5). Their fold and stability were tested by circular dichroism (Fig. S2A) and differential scanning calorimetry (Fig. S2B); all appeared folded at room temperature. All three constructs contained the receptor (Hur)-binding domain of PyoG (residues 1 to 255) and were protected from degradation by trypsin in intact cells (Fig. 2B and C), which indicated that these constructs translocated across the outer membrane. This is in accordance with previously published work, where a pyocin receptor binding domain was sufficient to protect the pyocin from degradation by extracellular trypsin added to live P. aeruginosa cells (9, 10). However, only full-length PyoG and PyoG^1–485^ were trypsin protected in spheroplasts (Fig. 2B and C), indicating that these two constructs were capable of translocating across the inner membrane. Trypsin protection of PyoG^1–485^ also implied that the cytotoxic domain of PyoG was not essential for inner membrane translocation, which, in contrast to a previous study, was suggested to be a requirement for colicin transport (11). PyoG^1–255^, which lacks the IMT domain, was not trypsin protected in P. aeruginosa spheroplasts. It was not possible to test if the IMT domain alone is sufficient for inner membrane transport, since PyoG^256-485^ could not be overexpressed. We conclude that the IMT domain is required for PyoG to cross the inner membrane.

10.1128/mbio.03396-21.2 FIG S2 Quality control of PyoG construct used for fluorescent labelling of P. aeruginosa. (A) CD spectra of 0.1 mg/mL PyoG-ImG (dashed line), PyoG (orange), PyoG^1–485^ (yellow), PyoG^31–485^ (blue), and PyoG^1–255^ (green) at room temperature in 20 mM NaF and 10 mM KPO_4_ buffer (pH 7). PyoG has a G4S linker and a cysteine at the C terminus. All other constructs have a cysteine directly added to the C terminus. An average from 9 measurements is shown. (B) DSC curve of 20 μM PyoG constructs in 50 mM Tris-HCl, pH 7, 150 mM NaCl, in the 25 to 60°C temperature range. The melting temperature of PyoG is 42.14 ± 0 and 49.56 ± 0.02°C, PyoG^1–485^ is 48.04 ± 0.14°C, and PyoG^1–255^ is 46.22 ± 0.02°C. All constructs have a melting curve similar to that of the wt PyoG-ImG, indicating that they are folded at room temperature. Download FIG S2, TIF file, 0.4 MB. Copyright © 2022 Atanaskovic et al. 2022 Atanaskovic et al. https://creativecommons.org/licenses/by/4.0/ This content is distributed under the terms of the Creative Commons Attribution 4.0 International license.

### Inner membrane translocation of PyoG requires FtsH and TonB1.

**FIG 3:**
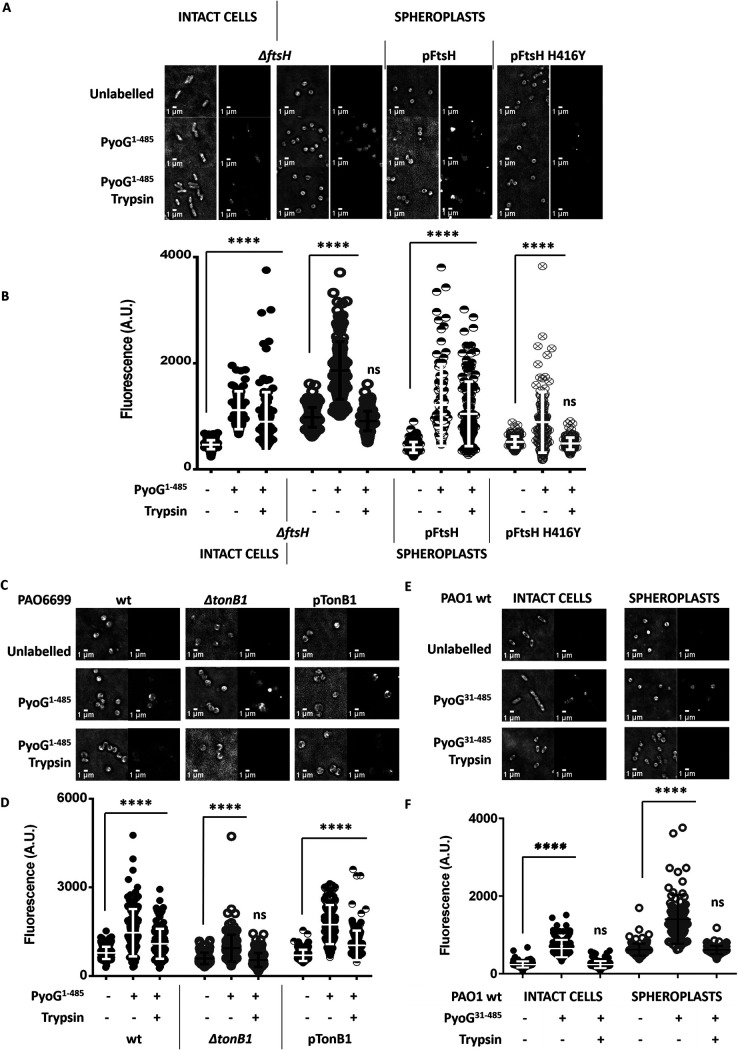
Inner membrane translocation of PyoG requires FtsH and binding to TonB1. PyoG^1–485^ is conjugated to AF488 via a C-terminal cysteine and added to P. aeruginosa spheroplasts at 2 μM. Nontranslocated pyocin is removed by trypsin treatment. (A and B) FtsH is required for inner membrane translocation of PyoG. PyoG is trypsin protected in intact Δ*ftsH* cells but not in Δ*ftsH* spheroplasts. This phenotype is complemented with FtsH expressed from a plasmid (pFtsH). No PyoG translocation is measured if the protease activity of FtsH has been disrupted with a point mutation (FtsH H416Y). (C and D) Labeling of Δ*tonB1* spheroplasts with fluorescent PyoG. No residual labeling is measured after trypsin treatment, which indicates that TonB1 is required for inner membrane translocation of PyoG. PyoG import into spheroplasts is restored if TonB1 is expressed from a plasmid (pTonB1). (E and F) The TonB binding box of PyoG, located in the unstructured N terminus, is required for PyoG import. PyoG^31–485^ lacks the first 30 residues of PyoG and is conjugated to AF488 via a cysteine in the C terminus. This deletion disrupts both the outer and the inner membrane import of PyoG, since the PyoG^31–485^ label is not trypsin protected in both intact cells and P. aeruginosa spheroplasts. (A, C, E, and G) Representative micrographs before and after trypsin treatment are shown. All snapshots were adjusted to the same intensity scale. (B, D, F, and H) Average fluorescence intensities were measured for 150 cells per condition. Means from three biological replicates with standard deviations are shown. Fluorescence intensities for labeled and trypsin treated groups under each condition are compared to the unlabeled control. ****, *P* value below 0.0001 in the Kruskal-Wallis Test; ns represents no significant difference or lack of fluorescent labeling.

PyoG killing activity has previously been linked to Hur, the outer membrane receptor, TonB1, the inner membrane protein that provides energy for outer membrane translocation, and FtsH, in the inner membrane (5). Since FtsH is not essential in P. aeruginosa, we used PAO1 as a model organism to test if FtsH is required for outer and/or inner membrane translocation of PyoG. PAO1 wild-type (wt) and Δ*ftsH* cells were exposed to fluorescent PyoG^1–485^ and to trypsin, as described for Fig. 2. PyoG^1–485^ was trypsin protected in Δ*ftsH* intact cells (Fig. 3A and B), demonstrating that outer membrane translocation of PyoG does not require FtsH. On the other hand, PyoG^1–485^ was not trypsin protected when added to Δ*ftsH* spheroplasts (Fig. 3A and B), indicating that FtsH was required for inner membrane translocation of PyoG. Additionally, when the *ftsH* deletion was complemented from a plasmid, PyoG killing activity (Fig. S3A) and inner membrane translocation (Fig. 3A and B) were restored. Complementation with FtsH H416Y, a mutant of FtsH that does not have protease activity (18), did not restore PyoG killing activity (Fig. S3A) or inner membrane translocation (Fig. 3A and B). Therefore, proteolytically active FtsH is required for PyoG to cross the inner membrane.

10.1128/mbio.03396-21.3 FIG S3 Plate killing assay of P. aeruginosa complemented strains. (A) PAO1 Δ*ftsH* strain complemented with FtsH expressed from a plasmid (pFtsH) or a protease inactivated version of FtsH (pFtsH H416Y). Only complementation with wt FtsH restored PyoG sensitivity. (B) P. aeruginosa PAO6699 Δ*tonB1* strain was complemented with TonB1 expressed from a plasmid (pTonB1). Pyocin sensitivity is restored in this strain. Download FIG S3, TIF file, 0.3 MB. Copyright © 2022 Atanaskovic et al. 2022 Atanaskovic et al. https://creativecommons.org/licenses/by/4.0/ This content is distributed under the terms of the Creative Commons Attribution 4.0 International license.

We also tested if TonB1, a protein previously linked to pyocin outer membrane translocation (9, 10), is required for PyoG import into spheroplasts. This protein was required for inner membrane translocation of PyoG, since PyoG^1–485^ was not trypsin protected in Δ*tonB1* spheroplasts (Fig. 3C and D). Complementation of Δ*tonB1* strain with TonB1 expressed from a plasmid restored PyoG killing activity (Fig. S3B) and inner membrane import (Fig. 3C and D). We also identified a region of PyoG that is involved in binding to TonB1 (Fig. S4) and demonstrated that this region is required for both outer and inner membrane import (Fig. 3E and F). Pyocins S5 (9) and S2 (10) bind TonB1 via a TonB box located in the unstructured N terminus (TonB box of S5 is S^10^MV^12^ and of S2 is M^11^VITH^15^). Even though the first 30 residues of PyoG share no sequence homology to pyocins S2 and S5, this region of PyoG is also predicted to be unstructured (5). Therefore, we deleted the first 30 residues in the unstructured N terminus of PyoG to test if this deletion affects TonB1 binding. The deletion, which renders PyoG inactive against P. aeruginosa (Fig. S4C), disrupted binding of PyoG to TonB1 (Fig. S4A), but it did not affect binding to the receptor Hur (Fig. S4B), confirming that the TonB box of PyoG is in the unstructured N terminus. Unlike PyoG^1–485^, PyoG^31–485^ did not translocate into intact cells or spheroplasts (Fig. 3E and F), demonstrating that the TonB box is required for both the outer and the inner membrane translocation steps.

10.1128/mbio.03396-21.4 FIG S4 Deletion of the first 30 residues of PyoG disrupts TonB1 binding and the pyocin killing activity. (A and B) Pulldowns with the PyoG^31–485^ and PyoG^1–485^ constructs. This construct was used for fluorescent labeling of P. aeruginosa (Fig. 3G and H). It has a cysteine and a His_6_ tag in the C terminus and was used as bait protein in pulldown assays. Periplasmic TonB1 (A) and Hur (B) were purified as described previously (5). Both proteins had no purification tag and were used as prey proteins. All proteins were mixed to a final concentration of 10 μM in binding buffer (50 mM Tris-HCl, pH 7.8, 250 mM NaCl; 1% β-OG was added if Hur was used) and bound to nickel beads at room temperature. Unbound protein was washed in the same buffer, and bound proteins were eluted in the presence of 250 mM imidazole. Eluate was analyzed on 12% SDS-PAGE gels. Proteins that were added to beads are indicated above each lane. Positions of proteins are labeled on the right side of each gel. PyoG^31–485^ does not bind TonB1 in the pulldown assay (A), but it binds to Hur (B). This indicates that the first 30 residues of PyoG contain the TonB1 binding box and that this region is not essential for receptor binding. (C) The killing activity of PyoG, which lacks the first 30 residues (PyoG^Δ1-30^). Unlike PyoG^31–485^, PyoG^Δ1-30^ contains the cytotoxic domain, and its activity can be tested in a plate killing assay. Three-microliter drops of 10 μM wt and Δ1-30 PyoG were spotted on PAO1 lawns. The deletion of the first 30 residues of PyoG disrupted its killing activity against PAO1, which indicates that this region of the pyocin is essential for its import into bacterial cells. Download FIG S4, TIF file, 1.2 MB. Copyright © 2022 Atanaskovic et al. 2022 Atanaskovic et al. https://creativecommons.org/licenses/by/4.0/ This content is distributed under the terms of the Creative Commons Attribution 4.0 International license.

### Model of nuclease bacteriocin transport across the inner membrane.

**FIG 4:**
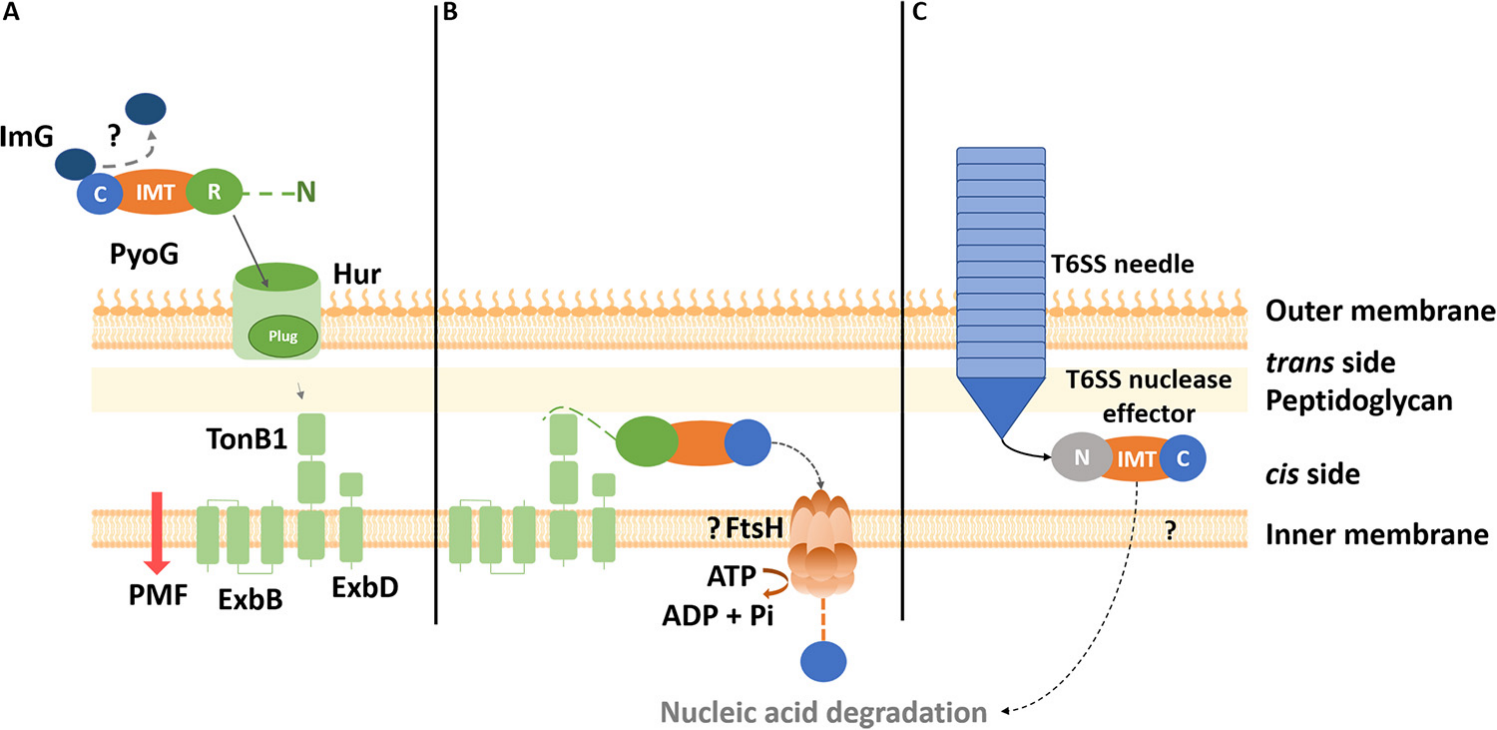
Probable import mechanism of nuclease pyocin G and a T6SS effector. (A) PyoG uses Hur, a TBDT, as its outer membrane receptor and translocator (5). The pyocin is composed of an unstructured N terminus (dashed line), a receptor binding domain (R), a conserved inner membrane translocation domain (IMT domain), and a cytotoxic nuclease domain (C). Like in the case of nuclease colicins (29), the immunity protein (Im) probably disassociates from the C domain during translocation. The plug domain of Hur interacts with the periplasmic domain of TonB1 (5). The binding of TonB1 and the pyocin R domain to the TBDT induces a conformational change that dislocates the plug of the TBDT, allowing the passage of the pyocin through the TBDT (10). The TonB box, located in the N terminus of PyoG, is essential for this translocation step, probably because TonB1 pulls PyoG through Hur and into the periplasm (9, 10). (B) Translocation of PyoG from the periplasm into the cytoplasm also requires TonB1 binding. The exact role of TonB1 in inner membrane transport remains unknown. Potentially, TonB1 positions the pyocin on the surface of the inner membrane so it can interact with other proteins involved in inner membrane transport. This translocation step requires the IMT domain, a conserved domain present in all nuclease bacteriocins. Inner membrane transport of PyoG requires FtsH, an inner membrane ATPase/protease previously associated with the killing activity of PyoG (5) and nuclease colicins (10). FtsH must be proteolytically active for PyoG to translocate into the cytoplasm. Inner membrane transport of PyoG could be coupled with FtsH-dependent proteolytic processing that releases the C domain into the bacterial cytoplasm, as previously demonstrated for nuclease colicins (15, 19). (C) Like nuclease bacteriocins, nuclease T6SS effectors contain the IMT domain (C, cytotoxic domain; IMT, inner membrane translocation domain; N, other domains and motifs in the N terminus). The needle and needle tail proteins can deliver these effectors to the *cis* side of the peptidoglycan layer or directly to the bacterial cytoplasm (26). Inner membrane proteins required for the inner membrane transport of T6SS effectors are currently unknown. It is possible that, as in the case of nuclease PyoG, this translocation step depends on the IMT domain.

The present study shows that the IMT domain, TonB1, and the ATPase/protease FtsH are all essential for the inner membrane translocation step of PyoG. Taken together with previous studies that demonstrate FtsH-dependent processing of nuclease colicins during import (13, 19), we propose a model of PyoG inner membrane translocation (Fig. 4). The PyoG receptor binding domain (residues 1 to 255) binds to Hur, which in turn interacts with TonB1 via its plug domain. The TonB box in the bacteriocin N terminus then associates with TonB1, likely pulling the toxin through the TBDT to the periplasm in its entirety (Fig. 4A) (10). PyoG associates with TonB1 via its TonB box, but whether this interaction also exploits the PMF dependence of TonB1 or other interaction partners is currently unknown. One possible role of the TonB1 interaction may be to localize PyoG close to the inner membrane from where the IMT domain either interacts directly with the membrane or even with FtsH itself for transfer across the membrane and proteolytic processing (13, 19). However, we were unable to demonstrate a direct interaction between FtsH and PyoG by pulldown assay (data not shown). It is also uncertain whether the entire IMT domain region of PyoG gets imported into the cytoplasm. Previous colicin studies suggest that colicins D, E3 (19), E2, and E7 (13) undergo proteolytic processing during import. A cleavage site positioned within the IMT domain was defined for these colicins. Other studies on colicins have suggested that direct interaction of the transported nuclease with the cytoplasmic membrane is a requirement for transport to the cytoplasm (20, 21). However, PyoG constructs lacking the nuclease are still able to translocate (Fig. 2). The IMT domain was sufficient to initiate inner membrane translocation, and potentially this domain also inserts into the inner membrane. Additionally, it is possible that the IMT domain initiates inner membrane transport through a mechanism involving other inner membrane proteins. The IMT domain of the nuclease colicin ColD has previously been shown to interact with inner membrane proteins essential for the ColD killing activity. The IMT domain of colicin D, which shares 26% sequence identity with that of PyoG, has been shown to bind TonB (22, 23) as well as the signal peptidase, LepB, in the inner membrane. Therefore, it is possible that the PyoG IMT domain also has additional interaction partners in the inner membrane.

We showed that some T6SS effectors, such as the ECA1669 protein from Erwinia carotovora (PDB entry 3MFB), share structural similarities with nuclease bacteriocins (Fig. 1A). Both groups of toxins can have an HNH motif in the nuclease domain and the conserved IMT domain upstream of the nuclease domain (Fig. 1). Therefore, nuclease bacteriocins and T6SS effectors may share similarities in their inner membrane translocation mechanism. T6SS effectors are loaded to the T6SS needle or needle tail proteins, which penetrate the outer membrane, reach the *cis* side of the peptidoglycan layer (24, 25), and deliver the effector to the bacterial cell (26). While the T6SS effectors are directly delivered to the *cis* side of the peptidoglycan layer or to the bacterial cytoplasm, bacteriocins may require the pulling force of TonB1 to reach this cell envelope compartment in a mechanism similar to the one exerted by TolA on the TolB-Pal complex (27). The specifics of T6SS effector inner membrane translocation are unknown. Although the sequence of the IMT domain is divergent between different T6SS effectors and bacteriocins, the main structural features are conserved (Fig. 1A), suggesting both toxin groups share similarities in their inner membrane translocation mechanism. It is possible that, as in the case of PyoG, the IMT domain of T6SS effectors is required for inner membrane translocation (Fig. 4C). Potentially, this translocation also requires FtsH, but this has yet to be tested experimentally.

The conserved IMT domain has previously been linked to bacteriocin killing activity (6), but until now its role in bacteriocin import has been unknown. In this study, we demonstrate that the IMT domain is specifically required for inner membrane translocation of nuclease bacteriocins. We also found this domain in T6SS nuclease effectors, which offers a link between nuclease effector import in contact-dependent and -independent competition systems. While these systems use different mechanisms to deliver cytoplasmic effectors to the bacterial periplasm, the transport of the effector across the inner membrane may be conserved.

## MATERIALS AND METHODS

### Sequence analysis of IMT domain-containing proteins.

IMT domain (PF06958.7)-containing proteins were identified in uniref100 using HMMer. Domains were identified using Pfam 27 with an E value of <1 × 10^−10^. Proteins were predicted to be T6SS effectors if they contained domains associated with T6SS transport (PAAR, Vgr, and Hcp) or shared significant homology with proteins from the SecRet6 T6SS effector data set (BLAST). Taxonomy of species with IMT domain was determined using NCBI Commontree and visualized using iTOL (28).

### Bacterial strains, media, and growth conditions.

All strains (Table 1) were cultured in LB (10 g/liter tryptone, 10 g/liter NaCl, 5 g/liter yeast extract, pH 7.2) or M9 medium (8.6 mM NaCl, 18.7 mM NH_4_Cl, 42.3 mM Na_2_HPO_4_, 22.0 mM KH_2_PO_4_, 0.4%, wt/vol, glucose, 2 mM MgSO_4_, 0.1 mM CaCl_2_) at 37°C with shaking (140 rpm). P. aeruginosa Δ*ftsH* strain was grown on salt-free LB medium. Δ*tonB1* mutant of P. aeruginosa and the parent strain PAO6609 were grown in LB medium supplemented with 100 μM FeCl_3_. P. aeruginosa Δ*hur* mutant was grown in the presence of 10 μg/mL tetracycline; 100 μg/mL carbenicillin and 25 μg/mL triclosan were used for selecting plasmid transformants of P. aeruginosa.

**TABLE 1:** List of strains used in this study

Species and strain	Characteristic(s)	Source or reference
Escherichia coli		
BL21(DE3)	Expression of His-tagged bacteriocins and TonB1	New England Biolabs
NEB5α	Plasmid propagation	New England Biolabs
S17-1	Conjugation with P. aeruginosa	American Type Culture Collection
Pseudomonas aeruginosa		
PAO1	Wild type	Washington Library (30)
Δ*hur*	PAO1 with a transposon insertion in locus PA1302 (*hur*::IS*lacZ*/*hah*); library ID PW3356	
PAO1 Δ*ftsH*	*ftsH* deletion mutant	31
pFtsH	Δ*ftsH* mutant of PAO1 complemented with pREN144	This study
pFtsH H416Y	Δ*ftsH* mutant of PAO1 complemented with pREN145	
PAO6609	*met-9011 amiE200 strA pvd-9*	32
ΔtonB1	*tonB1*::ΩHg transposon mutant in PAO6609 background	
pTonB1	*ΔtonB1* mutant of PAO6609 complemented with ptonB1	This study

### Plasmids.

pET21a(+) was used as a backbone for the expression of ColD and PyoG constructs in E. coli BL21(DE3). pMMB190 was used for complementation and gene expression in P. aeruginosa. All plasmids are listed in Table 2 and primers used for their construction in Table 3. Full-length PyoG, with the addition of a G4S linker and a cysteine in the C terminus in an operon with the ImG immunity protein, was synthesized by Genewiz and cloned into pET21a(+). pNGH262 (5) was used as the backbone for the construction of plasmids carrying PyoG constructs for the fluorescent labeling of P. aeruginosa. pG^1–485^-Cys was constructed by PCR, amplifying the first 485 residues of PyoG and by adding a cysteine at the C terminus of the construct by PCR mutagenesis (5). pG1-255-Cys was constructed by introducing a cysteine and an XhoI site after residue A255 in pNGH262 and cutting out the region between the two XhoI sites. Genes used for P. aeruginosa complementation (*ftsH*, *ftsH* H416Y, and *tonB1*) were synthesized by Genewiz and restriction cloned into pMMB190 using the EcoRI and HindIII sites. Plasmid DNA was isolated from NEB5α cultures grown at 37°C in LB with the appropriate antibiotic, using a Monarch Plasmid Miniprep kit (NEB). Sequencing was through the company Genewiz.

**TABLE 2:** List of plasmids used in this study

Plasmid	Insert	Vector	Description	Source or reference
pET21a(+)			pBR322 origin, His tag, Amp^r^	NEB
pMMB190			Broad-host-range cloning vector, Amp^r^, pMMB66EH, *tac* promoter, LacZα	33
pNGH262	PyoG, ImG-His_6_	pET21a(+)	His_6_-Im-PyoG cloned at *NdeI* and *HindIII* sites	5
pNGH263	ImG-His_6_	pACYCDuet1	PyoG immunity protein cloned at NdeI and XhoI	5
pG-G4S-Cys	PyoG-G4SC, ImG-His_6_	pET21a(+)	PyoG with a G4S linker and cysteine in the C terminus	This study
pG^1–485^-Cys	PyoG^1–485^Cys-His_6_	pET21a(+)	PyoG^1–485^-Cys-His_6_ cloned at NdeI and HindIII sites	This study
pG1-255-Cys	PyoG^1–255^Cys-His_6_	pET21a(+)	PyoG^1–255^-Cys-His_6_ cloned at NdeI and HindIII sites	This study
pGΔ1-30	PyoG^Δ1-30^, ImG-His_6_	pET21a(+)	DNA encoding His_6_-Im-PyoG, lacking the first 30 residues of PyoG, cloned at *Nde*I and *Hind*III sites	This study
pG3^1–485^-Cys	PyoG^31–485^Cys-His_6_	pET21a(+)	DNA encoding PyoG^31–485^-Cys-His_6_ cloned at NdeI and HindIII sites	This study
pREN144	His6-TEV-FtsH	pMMB190	*ftsH* from PAO1 with an N-terminal His6 and TEV site	This study
pREN145	His6-TEV-FtsH, point mutation H416Y	pMMB190	*ftsH* H416Y from PAO1 with an N-terminal His6 and TEV site	This study
pTonB1	*tonB1*	pMMB190	*tonB1* from PAO1 genome	This study

**TABLE 3:** List of primers used in this study

Primer	Sequence (5′→3′)	Description
G485-Cys-F	AAAAAACTCGAGTGTGTTAAACATGACGTACACAGGTTTG	Cloning of PyoG^1–485^-Cys from pNGH262 for the construction of pG^1–485^-Cys
G485-Cys-R	AAAAAACATATGGCACGTCCGATTG	
G255-Cys-Xho-F	CACCGAGCCGTTCTCGAGACAAGCTGGCATGGC	Introduction of Cys and XhoI site after codon A255 of PyoG for the construction of pG1-255-Cys
G255-Cys-Xho-R	CCATGCCAGCTTGTCTCGAGAACGGCTCGGTGG	
G30-Nde-F	GGTGGTGGCACGCATATGGGTATTGGTCCGATC	Introduction of NdeI site after codon T30 of PyoG for the construction of pGΔ1-30 and pG3^1–485^-Cys
G30-Nde-R	CGGACCAATACCCATATGCGTGCCACCACC	
FtsH-F	AAAAAAGGATCCACGGGCGAGGGTTCATAAAG	Cloning of *ftsH* from PAO1 genomic DNA into pMMB190 for the construction of pREN144 and pREN145
FtsH-R	AAAAAAGCTTAATCGGGGGTGACATTGAGG	

### Conjugation of P. aeruginosa.

Chemically competent E. coli S17-1 cells were prepared by CaCl_2_ treatment. A volume of 50 mL of overnight culture, grown at 37°C in LB, was pelleted on 5,000 × *g* for 10 min. Cells were resuspended in 50 mL ice-cold 0.1 M CaCl_2_ and kept on ice for 30 min. Cells were then pelleted again, resuspended in 1 mL 0.1 M CaCl_2_, and incubated on ice for 5 more min before transformation. Competent cells were transformed by heat shock. A volume of 50 μL of competent cells was mixed with 5 μL of DNA. Cells were incubated on ice for 30 min, shocked for 30 s at 42°C, and then incubated on ice for another 10 min. Cells were then plated on LB agar with antibiotics. P. aeruginosa PAO1 was transformed via conjugation with E. coli S17-1 carrying a plasmid of interest. PAO1 was grown overnight at 42°C and S17-1 at 37°C, with shaking, in LB medium. A volume of 2 mL of each culture was pelleted at 5,000 × *g*, 10 min. Each strain was then resuspended in 100 μL of LB medium, and the two strains were mixed together. The entire mix was then spotted on top of an antibiotic-free LB agar plate. The plate was incubated at 37°C for 4 h. The lawn was then scraped from the plate and resuspended in 1× phosphate-buffered saline (PBS), pH 7. The suspension was serially diluted in PBS. A volume of 100 μL of each dilution was plated on LB agar with 25 μg/mL triclosan for selecting against S17-1 and 100 μg/mL carbenicillin for selecting against untransformed PAO1.

### Expression and purification of bacteriocin constructs.

PyoG was expressed in complex with the His-tagged immunity protein ImG. Two copies of ImG were used, one in an operon with PyoG (pNGH262) and one on a separate plasmid (pNGH263), as described previously (5). PyoG^1–485^ and PyoG^1–255^ were expressed with an N-terminal His tag. E. coli BL21(DE3) was used for heterologous pyocin expression, as described previously (5).

### CD spectroscopy.

Proteins were dialyzed into 10 mM potassium phosphate, pH 8, 20 mM NaF and diluted to 0.1 mg/mL. Circular dichroism (CD) spectra were obtained using a Jasco J-815 spectropolarimeter over a wavelength range of 260 to 190 nm, a digital integration time of 1 s, and a 1-nm bandwidth. CD data in millidegrees were converted to mean residue ellipticity by dividing by molar concentration and number of peptide bonds.

### DSC.

The melting temperature of proteins, as an indication of their integrity, was determined by differential scanning calorimetry (DSC) performed on Malvern VP capillary DSC by David Staunton, Molecular Biophysics Suite, Department of Biochemistry, University of Oxford. Pyocin constructs were tested at 20 μM in 10 mM potassium phosphate, pH 8, 20 mM NaF.

### Conjugation of maleimide fluorophores to proteins.

Pyocins were fluorescently labeled using Alexa Fluor 488 C5 maleimide (AF488) fluorophore that was linked to proteins via an engineered C-terminal cysteine, as previously described (5).

### Labeling of live P. aeruginosa cells with fluorescent pyocins.

Fluorescent PyoG constructs conjugated to AF488 were used to label P. aeruginosa. Bacteria were grown overnight in M9 medium at 37°C with shaking. A volume of 1 mL of this overnight culture was pelleted and resuspended in 10 mL M9 medium and grown until an optical density at 600 nm (OD_600_) of 0.5. All pelleting steps were performed at 7,000 × *g* for 3 min. A volume of 1 mL of cells was washed in PBS, pH 7, and labeled with 2 μM pyocin for 30 min at room temperature. The unbound pyocin was removed by three washes in PBS. For the trypsin protection assay, after labeling, cells were exposed to 0.5 mg/mL trypsin for 1 h at 30°C in PBS with 35 μg/mL chloramphenicol. After a final washing step, bacteria were resuspended in 30 μL of PBS. A volume of 3 μL of cells was then loaded onto agarose pads, prepared using Geneframes (Thermo Scientific). A volume of 80 μL of 1% (wt/vol) agarose in PBS was pipetted into the Geneframe (17 by 28 mm). The surface was flattened with a cover slip and excess agar removed. Once the agar solidified the cover slip was removed, the bacterial suspension added, and a new coverslip attached to the adhesive side of the Geneframe.

### Labeling of P. aeruginosa spheroplasts with fluorescent pyocins.

Bacteria were grown overnight in M9 medium at 37°C with shaking. A volume of 1 mL of this overnight culture was pelleted and resuspended in 10 mL M9 medium and grown until an OD_600_ of 0.5. All pelleting steps were performed at 3,000 × *g* for 10 min. A volume of 1 mL of cells was pelleted and resuspended in PBS, pH 7, with the addition of 0.5 M sucrose, 20 mM EDTA, and 1.5 mg/mL lysozyme and incubated for 45 min on room temperature. Cells were then washed into PBS, 0.5 M sucrose and mixed with 2 μM fluorescent pyocin. After 30 min of incubation at room temperature, unbound pyocin was removed by three washes in PBS, 0.5 M sucrose. For the trypsin protection assay, after labeling, cells were exposed to 0.5 mg/mL trypsin for 1 h at 30°C in PBS, 0.5 M sucrose. After a final washing step, bacteria were resuspended in 30 μL of PBS, 0.5 M sucrose and loaded onto agar pads as described above. Agar was supplemented with 0.5 M sucrose to prevent the bursting of the spheroplasts.

### Image collection and data analysis.

All images were collected on an Oxford Nanoimager S microscope. In the case of PyoG constructs, images were collected at 100-ms exposure and 20% 488-nm laser power. For every image, 20 frames were collected and merged using the command “Zproject” in Image J. Average fluorescence was measured for a total of 50 cells per condition per repeat and was corrected by subtracting the average background fluorescence. All experiments were conducted in 3 biological repeats. Fluorescence intensity of experimental groups (groups exposed to fluorescent pyocin) was compared to the unlabeled control by the Kruskal-Wallis test using Dunn’s test as the *post hoc* procedure (confidence level, 0.001). The analysis was performed by GraphPad Prism version 6.04 for Windows, GraphPad Software, La Jolla, California, www.graphpad.com.

### Plate killing assays.

P. aeruginosa was grown in LB at 37°C to an OD_600_ of 0.6. Bacterial lawns were prepared by addition of 250 μL of culture to 5 mL of molten soft LB-agar (0.75% [wt/vol] agar in LB) and were poured over LB-agar plates. Once set and dry, 3 μL of 3-fold serially diluted pyocins, ranging from 10 μM to ∼57 pM, was spotted on top of the lawn. Lawns were grown overnight at 37°C and cytotoxicity was determined by observation of clearance zones.
