# Supplementary figures and images for: Bacterial Competition Systems Share a Domain Required for Inner Membrane Transport of the Bacteriocin Pyocin G from Pseudomonas aeruginosa
Source: mBio. 2022 Mar 28;13(2):e03396-21. doi: 10.1128/mbio.03396-21 (PMC9040868; doi:10.1128/mbio.03396-21)

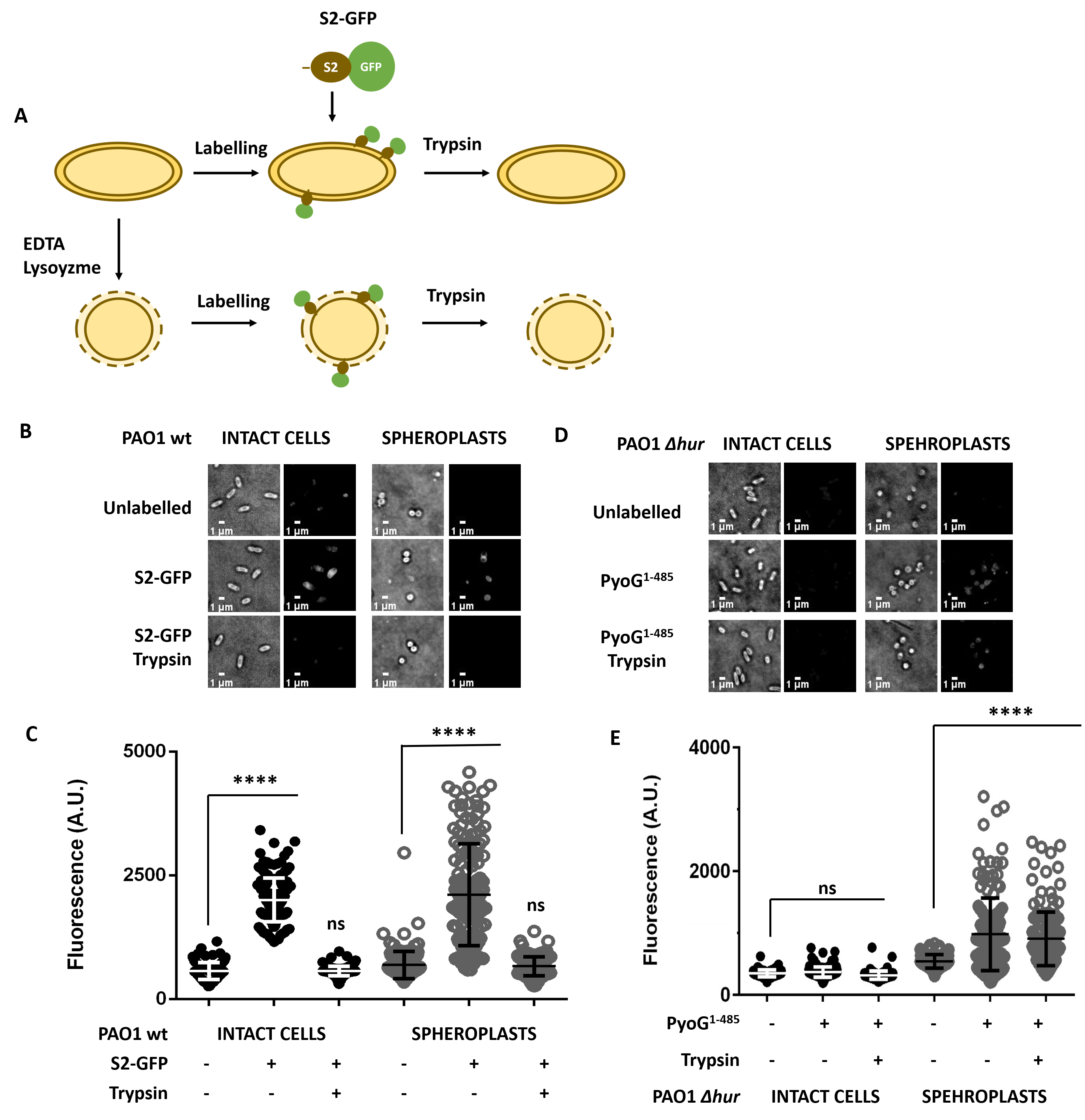

Supplement: FIG S1 [file mbio.03396-21-sf001.tif]

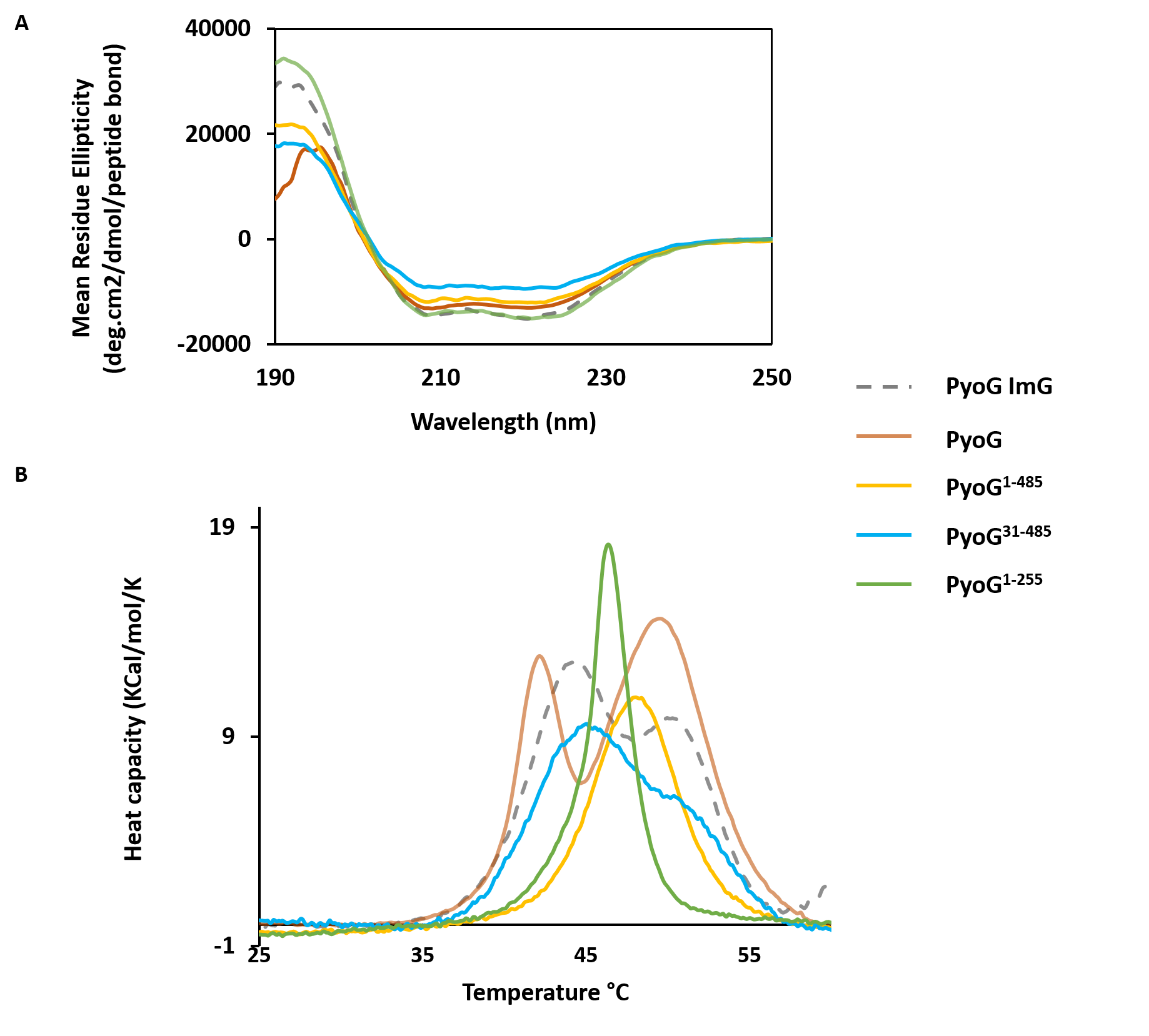

Supplement: FIG S2 [file mbio.03396-21-sf002.tif]

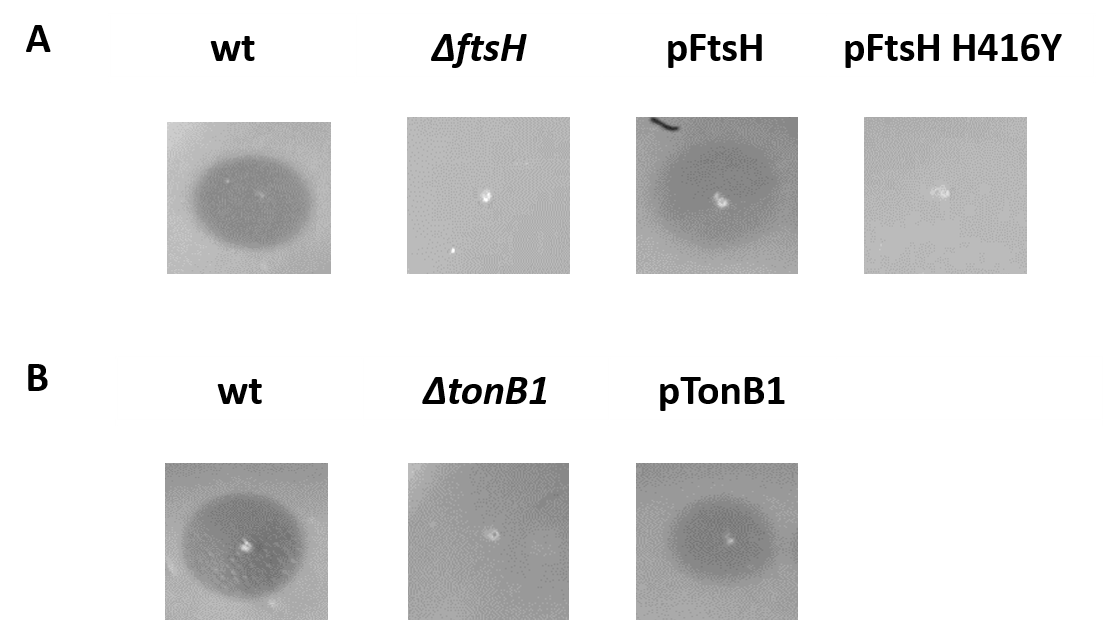

Supplement: FIG S3 [file mbio.03396-21-sf003.tif]

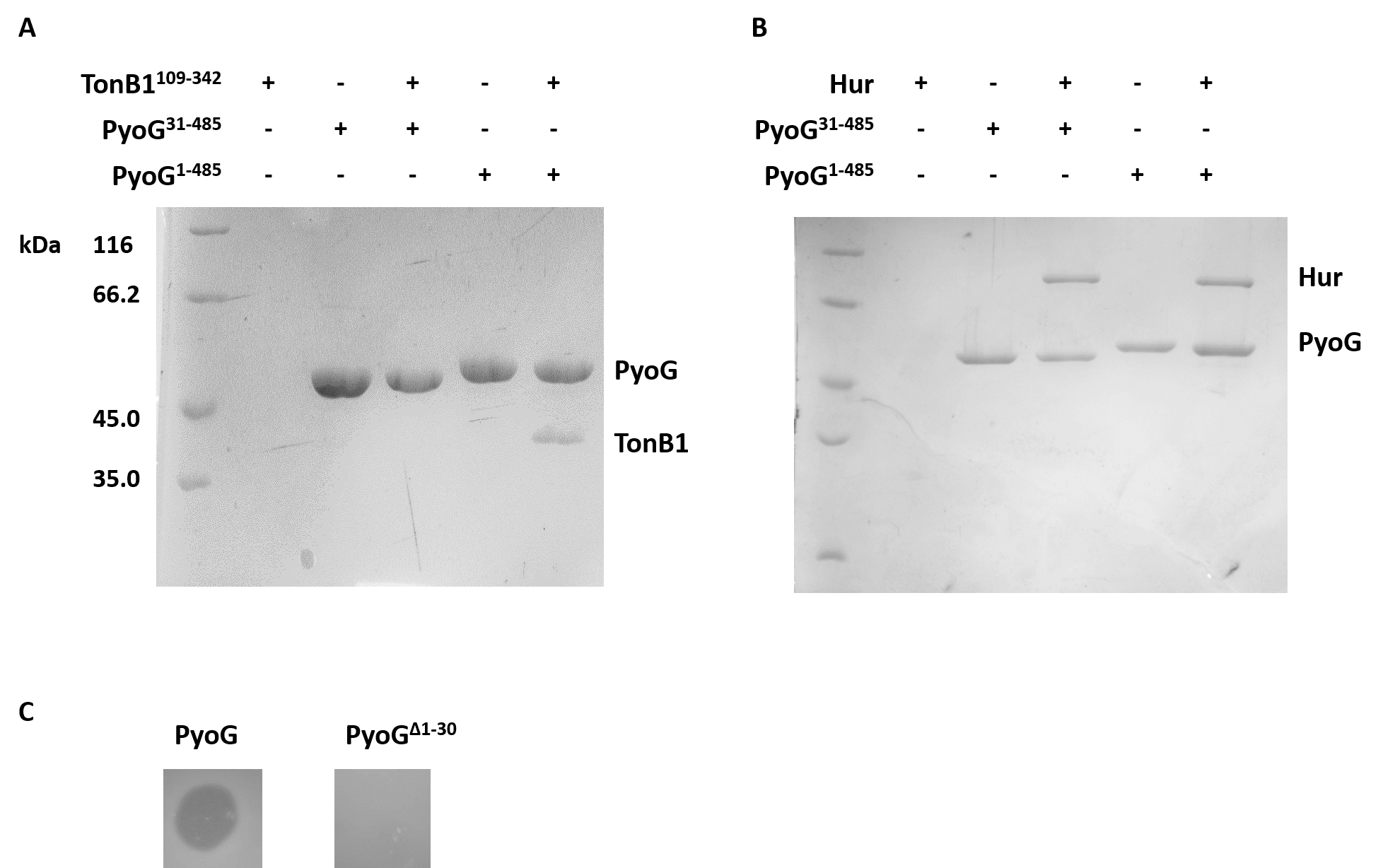

Supplement: FIG S4 [file mbio.03396-21-sf004.tif]
